# Aminaphtone Efficacy in Primary and Secondary Raynaud’s Phenomenon: A Feasibility Study

**DOI:** 10.3389/fphar.2019.00293

**Published:** 2019-04-04

**Authors:** Barbara Ruaro, Carmen Pizzorni, Sabrina Paolino, Elisa Alessandri, Alberto Sulli

**Affiliations:** Research Laboratory and Academic Division of Clinical Rheumatology, Department of Internal Medicine, University of Genova, IRCCS San Martino Polyclinic Hospital, Genova, Italy

**Keywords:** Raynaud phenomenon, aminaphtone, blood perfusion, systemic sclerosis, laser speckle contrast analysis, microcirculation, clinical symptoms, Raynaud condition score

## Abstract

**Objectives:**

The aim of this six-month open feasibility study was to evaluate skin blood perfusion and clinical symptom changes during aminaphtone treatment in patients with either primary or secondary Raynaud’s phenomenon to systemic sclerosis.

**Methods:**

Ninety-two patients referring for Raynaud’s phenomenon have been enrolled in November during routine clinical assessment, after informed consent. Aminaphtone was administered 75 mg twice daily in addition to current treatments to forty-six patients. Skin blood perfusion was measured by Laser Speckle Contrast Analysis (LASCA) at the level of fingertips, periungual areas, dorsum and palm of hands, and face at baseline (W0), after one (W1), four (W4), twelve (W12) and twenty-four (W24) weeks of treatment. Raynaud’s condition score (RCS) and both frequency and duration of Raynaud’s attacks were assessed at the same time.

**Results:**

Compared with the control group, despite colder period of the year, aminaphtone treated patients showed a progressive statistically significant increase of blood perfusion, as well as a decrease of RCS, frequency of Raynaud’s attacks/day and their duration, from W0 to W12 in all skin areas. From W12 to W24 no further increase of blood perfusion was observed. The results were similar in both primary and secondary Raynaud’s phenomenon patients. Five weeks after aminaphtone discontinuation blood perfusion values were significantly higher than those at baseline in the majority of skin areas.

**Conclusion:**

This study demonstrates that aminaphtone treatment increases skin blood perfusion and improves Raynaud’s phenomenon clinical symptoms, with sustained efficacy up to 6 months, even in patients with systemic sclerosis. A randomized, blind, controlled, clinical trial including a larger number of subjects is advisable to confirm these early results.

## Introduction

Raynaud’s phenomenon (RP) is a vasospastic disorder causing discoloration of fingers, toes, and occasionally other areas like nose and tongue, with classic triphasic expression: pallor (ischaemic phase), followed by cyanosis (cyanotic phase) and lastly redness (reactive hyperemic phase) (Herrick, 2012; Hughes and Herrick, 2016; Wigley and Flavahan, 2016). The pathogenesis of RP is still not entirely clear or understood, but recent insights into the pathogenic mechanisms underlying RP include vascular, neuronal and intravascular abnormalities which may identify crucial key points and potential targets for therapeutic intervention (Herrick, 2012; Hughes and Herrick, 2016; Wigley and Flavahan, 2016).

Raynaud’s phenomenon is classified as primary (idiopathic, not associated with any disease), or secondary to several clinical conditions, such as connective tissue diseases, in particular systemic sclerosis (SSc) (Bernero et al., 2013; Park et al., 2015).

Systemic sclerosis is characterized by a deregulation of the vascular tone, in which RP is the most frequent clinical manifestation, associated with structural damage and intimal thickening of the vascular wall, which leads to reduced blood flow and chronic tissue ischemia (Gabrielli et al., 2009; Cutolo et al., 2010b; Kanno et al., 2017). In addition, endothelial cell dysfunction, characterized by an imbalance between vasoconstrictor and vasodilator mediators, is a primary event in the pathogenesis of SSc, followed by fibrosis (Gabrielli et al., 2009; Sulli et al., 2009; Corallo et al., 2016; Hughes and Herrick, 2016).

In the last years, several clinical trials and observational studies on RP have been published, reflecting the increased awareness of the disease burden (Wigley et al., 1994, 1998; Fries et al., 2005; Thompson and Pope, 2005; Gliddon et al., 2007; Cutolo et al., 2014; Corallo et al., 2016). Pharmacological therapies for treatment and prevention of RP include calcium channel blockers, antiplatelet and anticoagulant drugs, endothelin receptor antagonist, phosphodiesterase inhibitors, iloprost, and statins (Wigley et al., 1994, 1998; Fries et al., 2005; Thompson and Pope, 2005; Gliddon et al., 2007; Abou-Raya et al., 2008; Herrick, 2013; Cutolo et al., 2014). However, current treatments for RP have limited efficacy, which was mainly demonstrated by physician/patient reported outcomes (PROs) (Wigley et al., 1994, 1998; Fries et al., 2005; Thompson and Pope, 2005; Gliddon et al., 2007; Abou-Raya et al., 2008).

Aminaphtone is a synthetic derivative of 4-aminobenzoic acid (2-hydroxy-3-methyl-1,4 apthohydroquinone-2-p-aminobenzoatate), which has been used for more than 40 years in some European and South American countries in the treatment of clinical consequences of microvascular impairment (e.g., chronic venous insufficiency of the lower limbs, ulcers of legs, and microangiopathy in diabetes) (De Anna et al., 1989; Pereira de Godoy, 2010; Belczak et al., 2014; Romano et al., 2014; Martinez-Zapata et al., 2016; Felice et al., 2018). Recently, aminaphtone was reported to improve the symptoms associated with RP, as well as to reduce endothelin-1 production on cultured human endothelial cells (Scorza et al., 2008a; Parisi et al., 2015).

Laser speckle contrast analysis (LASCA) is a validated technique that quantifies skin blood perfusion over an area (Ruaro et al., 2014, 2016; Sulli et al., 2014; Lambrecht et al., 2016).

The aim of this longitudinal six-month open feasibility study was to evaluate skin blood perfusion changes by LASCA and RP-related clinical symptoms by PROs during aminaphtone treatment, in patients with either primary RP or secondary RP to SSc.

## Materials and Methods

### Patients

Recruitment of all patients was performed in November 2016, during routine clinical assessment, at the outpatient clinic of the Division of Rheumatology of the University of Genova. Patients were enrolled in November to carry out the study during the six colder months of the year, in order to avoid that seasonal variations of temperature could influence skin blood perfusion assessment and study results. The study was conducted in accordance with the principles of the Declaration of Helsinki and Good Clinical Practice, and all patients provided written informed consent.

Forty-six consecutive patients with active RP, asking for treatment during standard clinical assessments, were recruited: 11 primary RP (mean age 49 ± 19 SD years, mean RP duration 6 ± 3 years) and 35 secondary RP to SSc according to the ACR/EULAR 2013 criteria (mean age 61 ± 17 SD years, mean RP duration 11 ± 9 years) (van den Hoogen et al., 2013; Wigley and Flavahan, 2016).

In patients with secondary RP, SSc duration was determined by onset of first non-Raynaud symptom clearly attributable to SSc (LeRoy et al., 1988; LeRoy and Medsger, 2001; Cutolo et al., 2007b). Furthermore, SSc patients were categorized as having limited (lcSSc) or diffuse cutaneous SSc (dcSSc) according to LeRoy criteria, as well as included into the proper pattern of microangiopathy by nailfold videocapillaroscopy and Cutolo’s criteria (13 patients showing the “Early,” 13 “Active,” and 9 “Late” pattern of microvascular damage) (LeRoy et al., 1988; Cutolo et al., 2000, 2004).

Patients with glucose-6-phosphate-dehydrogenase deficiency or other clinical conditions contraindicating the use of aminaphtone were excluded from the study.

Further 46 patients with RP (10 primary RP, mean age 56 ± 12 SD years, mean RP duration 8 ± 4 years; 36 secondary RP to SSc, mean age 63 ± 11 SD years, mean RP duration 12 ± 10 years) were also enrolled as a control group (nailfold videocapillaroscopy patterns: 14 “Early,” 13 “Active,” and 9 Late”).

### Aminaphtone Treatment and Concomitant Medications

Aminaphtone was administered 75 mg twice daily in addition to current treatments, as our usual clinical practice in RP patients, due to its ability to ameliorate the capillary resistance and permeability and to inhibit erythrocyte aggregation at microcirculation level (as reported inside technical sheet). The posology was in agreement with that reported inside the technical data sheet of the drug.

The exclusion criteria were: treatment with drugs that could potentially influence peripheral blood perfusion (iloprost, calcium channel blockers) and presence of recent digital ulcers requiring bosentan administration.

The inclusion criteria were: patients on stable drug regimen since at least 2 months prior to study entry, no changes made during the follow-up, treatment free period of at least 2 months from prostanoids and endothelin-1 receptor antagonists.

Concomitant treatments in patients treated with aminaphtone were: aspirin (average dosage 100 mg/die, used by 34 patients), proton pump inhibitors (30 patients), antihypertensive drugs (4 patients), cyclosporine (average dosage 150 mg/die, 6 patients), methotrexate (average dosage 10 mg/die, 4 patients). Concomitant treatments in control group were: aspirin (average dosage 100 mg/die, used by 34 patients), proton pump inhibitors (31 patients), antihypertensive drugs (6 patients), cyclosporine (average dosage 150 mg/die, 5 patients), methotrexate (average dosage 10 mg/die, 3 patients).

### Evaluation of Skin Blood Perfusion by Laser Speckle Contrast Analysis (LASCA)

Blood perfusion was measured as perfusion units (PU) in all patients by LASCA technique (PeriCam PSI, Perimed, Sweden), as previously reported (Ruaro et al., 2014, 2016; Sulli et al., 2014; Lambrecht et al., 2016), at the level of dorsal and palmar aspect of hands and face, at baseline (W0), after one (W1), four (W4), twelve (W12) and twenty-four (W24) weeks of aminaphtone treatment, and after one (W25) and five (W29) weeks since treatment discontinuation. Raynaud’s condition score (RCS) and both frequency and duration of Raynaud’s attacks were assessed at the same time (see below). For acclimatization, each patient stayed inside the building for a minimum of 15 min before the blood perfusion was examined, at room temperature of about 23°C.

**FIGURE 1:**
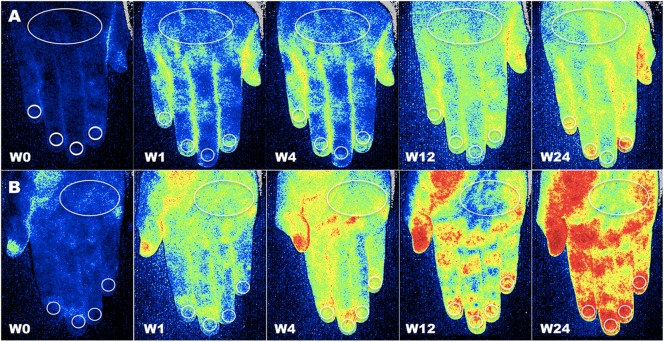
Assessment of blood perfusion. Example of evaluation of blood perfusion by LASCA technique at the level of dorsal **(A)** and palmar **(B)** aspect of hands, respectively, at baseline (W0), after one (W1), four (W4), twelve (W12) and twenty-four (W24) weeks of treatment with aminaphtone in patient F.B. (Blue color = low blood perfusion, yellow color = intermediate blood perfusion, red color = high blood perfusion (White circles = regions of interest for perfusion measuring at the level of dorsum, periungual, palm and fingertip areas).

After image recording, different regions of interest (ROIs) were drawn at the fingertip level, periungual areas, dorsum and palm of hands, tip of noise, and whole face (Sulli et al., 2014; Ruaro et al., 2016). Blood perfusion was measured inside the ROIs (see example in Figure 1). The evaluator was blind to both patient treatment and time of visit. For each anatomic area, the average BP was calculated by summing the perfusion values of the two sides, left and right.

All instrumental technical parameters were standardized for all patients and used at follow-up visits.

### Clinical Evaluation of Raynaud’s Symptoms

The clinical efficacy of aminaphtone on RP symptoms was evaluated at baseline (W0), after one (W1), four (W4), twelve (W12) and twenty-four (W24) weeks of treatment, and after one (W25) and five (W29) weeks since treatment discontinuation. The Raynaud condition score (RCS) that evaluates the limitation of daily activity on a scale from 1 to 10 (10 represents the total inability to do any activity) was used (Pope, 2011; Bose et al., 2015). Furthermore, the frequency (average number of event during the day) and duration (in minutes) of Raynaud’s attacks were also recorded.

### Nailfold Videocapillaroscopy (NVC)

All patients were assessed by nailfold videocapillaroscope (NVC), equipped with a 200× contact lens, connected to image analysis software (Videocap, DS MediGroup, Milan, Italy). Severity of microangiopathy was detected according to the proper pattern of microvascular damage (“Early,” “Active,” or “Late”), as previously reported (Cutolo et al., 2000, 2004; Sulli et al., 2008).

### Statistical Analysis

Statistical analysis was carried out by non-parametric tests. The Wilcoxon signed-rank test was used to compare paired groups of variables, and Mann–Whitney *U* test to compare unpaired groups of variables. Kruskal–Wallis test was used to compare continuous variables with nominal variables with more than two levels. Friedman test was employed to detect differences across multiple related comparisons. The *p* values lower than 0.05 were considered statistically significant.

## Results

Clinical characteristics of enrolled patients are reported in Table 1.

**Table 1:** Clinical findings in patients with Raynaud’s phenomenon.

		Total RP patients	Age (years)	Gender Female/Male	Weight (kg) PRP	RP duration (years)		NVC patterns Early/Active/Late	lcSSc/dcSSc
						PRP	SRP-SSc		
**AMI**	No. of patients	46		40/6		11	35	13/13/9	24/11
	mean ± SD		58 ± 11		65.1 ± 5.9	6 ± 3	11 ± 9		
**CNT**	No. of patients	46		40/6		10	36	14/13/9	25/11
	mean ± SD		60 ± 11		64.8 ± 6.3	7 ± 4	11 ± 10		


*AMI = aminaphtone treated group; CNT = control untreated group; RP = Raynaud’s phenomenon; PRP = primary Raynaud’s phenomenon; SRP = secondary Raynaud’s phenomenon; SSc = systemic sclerosis; NVC = nailfold videocapillaroscopy; Early, Active, Late = patterns of microangiopathy; lcSSc = limited cutaneous SSc; dcSSc = diffuse cutaneous SSc. AMI vs. CNT: *p* = not significant for all results*.

**FIGURE 2:**
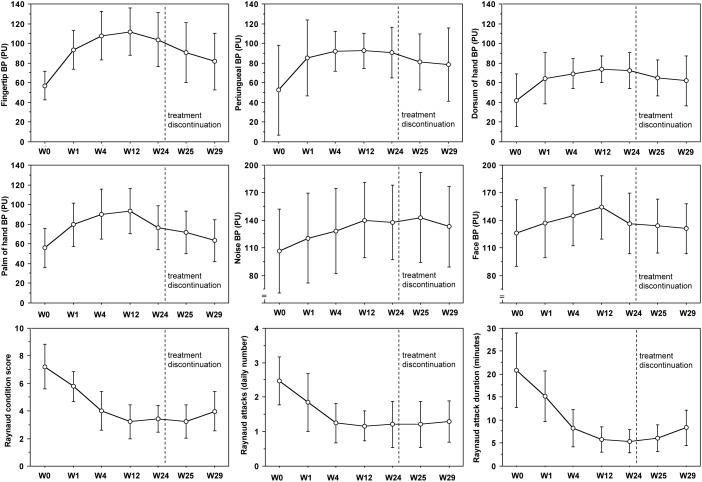
Blood perfusion and clinical trends during aminaphtone treatment. Variation of blood perfusion (BP) in six skin areas from baseline (W0) to twenty-four (W24) weeks during aminaphtone treatment, and after one (W25) and five (W29) weeks since treatment discontinuation, in all patients with Raynaud’s phenomenon. Variation of Raynaud Condition Score (RCS), Raynaud’s frequency (number of daily attacks) and duration (minutes) are also reported. Charts report means along with standard deviation values (PU = perfusion units). See Table 2 for statistical significances.

Despite colder period of the year, progressive statistically significant increase of blood perfusion was observed from W0 to W12 in all skin areas of RP patients (*p* < 0.001 for all skin areas) (see Figure 2 and Table 2A for perfusion values and statistical significance at single times). From W12 to W24 no further increase of blood perfusion was observed (Table 2A). Noteworthy was the fact that all patients on aminaphtone treatment had an increase of blood perfusion from W0 to W1; likewise 38/44 patients from W1 to W4, 36/43 from W4 to W12 and 8/43 patients from W12 to W24 had a further increase of blood perfusion.

**Table 2:** Variation of blood perfusion and clinical symptoms in RP patients.

		Fingertip BP PU mean ± SD	Periungueal BP PU mean ± SD	Palm BP PU mean ± SD	Dorsum BP PU mean ± SD	Noise BP PU mean ± SD	Face BP PU mean ± SD	RCS score 0–10 mean ± SD	RP frequency No. of daily attacks mean ± SD	RP duration minutes mean ± SD
**A. Cumulative RP patients treated with aminaphtone (46 patients)**		
**timing**	**Week 0**	57.1 ± 14.8	52.4 ± 35.6	56.1 ± 19.9	42.3 ± 26.7	106.5 ± 45.6	126.2 ± 36.1	7.22 ± 1.62	2.48 ± 0.69	20.83 ± 8.14
	**Week 1**	93.2 ± 19.4	85.0 ± 28.6	79.7 ± 21.9	64.5 ± 25.9	120.6 ± 48.7	137.1 ± 38.0	5.78 ± 1.09	1.85 ± 0.84	15.20 ± 5.52
	**Week 4**	107.7 ± 24.7	91.9 ± 20.2	90.2 ± 25.2	69.3 ± 15.1	128.4 ± 46.2	145.2 ± 33.2	4.00 ± 1.40	1.25 ± 0.58	8.25 ± 4.04
	**Week 12**	111.9 ± 24.3	92.3 ± 17.6	93.4 ± 23.0	73.8 ± 13.8	140.2 ± 41.1	154.0 ± 34.7	3.23 ± 1.23	1.16 ± 0.43	5.81 ± 2.79
	**Week 24**	103.81 ± 27.6	90.6 ± 25.7	76.7 ± 22.3	72.2 ± 18.4	137.5 ± 40.8	136.5 ± 32.9	3.42 ± 0.96	1.21 ± 0.67	5.40 ± 2.56
	**Week 25**	90.7 ± 30.3	81.2 ± 28.3	71.7 ± 21.9	64.7 ± 18.3	143.0 ± 49.2	134.0 ± 29.4	3.23 ± 1.19	1.21 ± 0.67	6.09 ± 2.98
	**Week 29**	81.7 ± 28.8	78.2 ± 37.2	63.3 ± 21.1	62.0 ± 25.3	133.1 ± 43.7	130.0 ± 27.4	3.98 ± 1.42	1.30 ± 0.60	8.37 ± 3.86
**Statistical significance**	W0 vs. W1	*p* < 0.0001	*p* < 0.0001	*p* < 0.0001	*p* < 0.0001	*p* = 0.0301	*p* = 0.0142	*p* < 0.0001	*p* < 0.0001	*p* < 0.0001
	W0 vs. W4	*p* < 0.0001	*p* < 0.0001	*p* < 0.0001	*p* < 0.0001	*p* = 0.0061	*p* = 0.0009	*p* < 0.0001	*p* < 0.0001	*p* < 0.0001
	W0 vs. W12	*p* < 0.0001	*p* < 0.0001	*p* < 0.0001	*p* < 0.0001	*p* = 0.0001	*p* < 0.0001	*p* < 0.0001	*p* < 0.0001	*p* < 0.0001
	W0 vs. W24	*p* < 0.0001	*p* < 0.0001	*p* < 0.0001	*p* < 0.0001	*p* = 0.0016	*p* = 0.0549	*p* < 0.0001	*p* < 0.0001	*p* < 0.0001
	W0 vs. W25	*p* < 0.0001	*p* < 0.0001	*p* < 0.0001	*p* < 0.0001	*p* = 0.0012	*p* = 0.0564	*p* < 0.0001	*p* < 0.0001	*p* < 0.0001
	W0 vs. W29	*p* < 0.0001	*p* < 0.0001	*p* = 0.0554	*p* < 0.0001	*p* = 0.0068	*p* = ns	*p* < 0.0001	*p* < 0.0001	*p* < 0.0001
	W1 vs. W4	*p* < 0.0001	*p* < 0.0001	*p* = 0.0016	*p* < 0.0001	*p* = 0.0007	*p* = 0.0007	*p* < 0.0001	*p* < 0.0001	*p* < 0.0001
	W1 vs. W12	*p* < 0.0001	*p* < 0.0001	*p* < 0.0001	*p* < 0.0001	*p* < 0.0001	*p* < 0.0001	*p* < 0.0001	*p* < 0.0001	*p* < 0.0001
	W1 vs. W24	*p* = 0.0305	*p* = ns	*p* = ns	*p* = 0.0361	*p* = 0.0247	*p* = ns	*p* < 0.0001	*p* < 0.0001	*p* < 0.0001
	W1 vs. W25	*p* = ns	*p* = ns	*p* = ns	*p* = ns	*p* = 0.0137	*p* = ns	*p* < 0.0001	*p* < 0.0001	*p* < 0.0001
	W1 vs. W29	*p* = 0.0032	*p* = ns	*p* = 0.0010	*p* = ns	*p* = ns	*p* = ns	*p* < 0.0001	*p* < 0.0001	*p* < 0.0001
	W4 vs. W12	*p* = 0.0005	*p* = 0.0188	*p* = 0.0519	*p* = 0.0002	*p* = 0.0031	*p* = 0.0011	*p* < 0.0001	*p* = 0.0441	*p* < 0.0001
	W4 vs. W24	*p* = ns	*p* = ns	*p* = 0.0014	*p* = ns	*p* = ns	*p* = ns	*p* = 0.0059	*p* = ns	*p* < 0.0001
	W4 vs. W25	*p* = 0.0002	*p* = 0.0469	*p* = 0.0005	*p* = ns	*p* = ns	*p* = 0.0175	*p* < 0.0001	*p* = ns	*p* = 0.0004
	W4 vs. W29	*p* < 0.0001	*p* = 0.0439	*p* < 0.0001	*p* = ns	*p* = ns	*p* = 0.0062	*p* = ns	*p* = ns	*p* = ns
	W12 vs. W24	*p* = ns	*p* = ns	*p* < 0.0001	*p* = ns	*p* = ns	*p* = 0.0050	*p* = ns	*p* = ns	*p* = ns
	W12 vs. W25	*p* < 0.0001	*p* = 0.0194	*p* < 0.0001	*p* = 0.0075	*p* = ns	*p* = 0.0002	*p* = ns	*p* = ns	*p* = ns
	W12 vs. W29	*p* < 0.0001	*p* = 0.0241	*p* < 0.0001	*p* = 0.0079	*p* = ns	*p* < 0.0001	*p* = 0.0001	*p* = 0.0125	*p* < 0.0001
	W24 vs. W25	*p* < 0.0001	*p* = 0.0030	*p* = ns	*p* = 0.0123	*p* = ns	*p* = ns	*p* = ns	*p* = ns	*p* = 0.0548
	W24 vs. W29	*p* < 0.0001	*p* = 0.0217	*p* < 0.0001	*p* = 0.0139	*p* = ns	*p* = ns	*p* = 0.0125	*p* = ns	*p* < 0.0001
	W25 vs. W29	*p* < 0.0001	*p* = ns	*p* = 0.0020	*p* = ns	*p* = 0.0465	*p* = ns	*p* < 0.0001	*p* = ns	*p* = 0.0002
**B. Cumulative RP untreated patients (controls) (46 patients)**		
	**Week 0**	72.9 ± 16.3	69.3 ± 16.4	62.2 ± 15.0	56.3 ± 12.8	130.9 ± 32.5	138.4 ± 39.1	3.83 ± 1.25	1.35 ± 0.71	6.39 ± 3.99
	**Week 24**	73.8 ± 19.0	70.0 ± 16.6	62.6 ± 15.3	58.1 ± 15.0	128.3 ± 28.2	138.1 ± 40.0	3.62 ± 1.03	1.33 ± 0.67	6.04 ± 3.44
	W0 vs. W24	*p* = ns	*p* = ns	*p* = ns	*p* = ns	*p* = ns	*p* = ns	*p* = ns	*p* = ns	*p* = ns


***A:** Variation of blood perfusion (BP), Raynaud Condition Score (RCS), Raynaud’s frequency (number of daily attacks) and duration (minutes) from baseline (W0) to week twenty-four (W24) during aminaphtone treatment, and after one (W25) and five (W29) weeks since treatment discontinuation, in patients with Raynaud’s phenomenon (RP). **B:** Trend of blood perfusion and clinical symptoms in not-treated Raynaud’s phenomenon patients. PU = perfusion units. Statistical significance between single times: Wilcoxon test*.

A progressive statistically significant decrease of RCS, frequency of Raynaud’s attacks/day and their duration was also recorded from W0 to W12 (*p* < 0.0001 for all) (see Figure 2 and Table 3A for clinical values and statistical significance at single times). From W12 to W24 clinical symptoms did not change significantly (Table 2A).

**Table 3:** Variation of blood perfusion and clinical symptoms splitted by primary or secondary RP.

		Fingertip BP PU mean ± SD	Periungueal BP PU mean ± SD	Palm BP PU mean ± SD	Dorsum BP PU mean ± SD	Noise BP PU mean ± SD	Face BP PU mean ± SD	RCS score 0–10 mean ± SD	RP frequency No. of daily attacks mean ± SD	RP duration minutes mean ± SD
**A. Primary RP patients treated with aminaphtone (11 patients)**		
**Timing**	**Week 0**	57.1 ± 12.3	53.0 ± 12.1	53.1 ± 7.9	42.9 ± 10.4	104.4 ± 38.7	128.0 ± 45.0	7.09 ± 1.14	2.27 ± 0.47	19.09 ± 7.01
	**Week 1**	88.1 ± 16.8	79.3 ± 28.9	75.6 ± 20.2	57.9 ± 21.7	122.0 ± 36.2	131.2 ± 27.2	5.64 ± 0.81	1.64 ± 0.50	16.36 ± 5.05
	**Week 4**	103.4 ± 24.8	90.7 ± 24.1	96.8 ± 39.0	67.3 ± 17.5	127.8 ± 36.1	145.3 ± 32.7	3.67 ± 1.41	1.11 ± 0.33	8.00 ± 3.00
	**Week 12**	110.8 ± 20.4	94.2 ± 21.2	106.34 ± 37.7	73.1 ± 14.8	146.6 ± 41.2	156.6 ± 38.2	3.33 ± 1.12	1.12 ± 0.34	6.67 ± 3.16
	**Week 24**	108.2 ± 32.0	100.8 ± 34.3	70.9 ± 29.1	76.7 ± 23.8	145.0 ± 30.7	129.7 ± 31.0	3.22 ± 1.09	1.13 ± 0.33	6.00 ± 3.00
	**Week 25**	102.9 ± 48.7	92.7 ± 37.6	78.0 ± 23.27	66.8 ± 19.7	157.7 ± 53.6	137.6 ± 28.7	3.33 ± 1.32	1.12 ± 0.32	7.33 ± 3.16
	**Week 29**	93.7 ± 52.5	77.2 ± 34.5	61.6 ± 29.4	62.7 ± 18.3	157.6 ± 51.4	131.7 ± 29.3	4.11 ± 2.15	1.22 ± 0.44	8.36 ± 2.65
**Statistical significance**	W0 vs. W1	*p* = 0.0005	*p* = 0.0217	*p* = 0.0012	*p* = 0.0269	*p* = 0.0186	*p* = ns	*p* = 0.0039	*p* = 0.0019	*p* = ns
	W0 vs. W4	*p* = 0.0007	*p* = 0.0020	*p* = 0.0057	*p* = 0.0038	*p* = 0.0558	*p* = ns	*p* < 0.0003	*p* = 0.0006	*p* = 0.0016
	W0 vs. W12	*p* = 0.0002	*p* = 0.0007	*p* = 0.0016	*p* = 0.0008	*p* = 0.0057	*p* = 0.0558	*p* < 0.0001	*p* = 0.0006	*p* = 0.0017
	W0 vs. W24	*p* = 0.0016	*p* = 0.0039	*p* = 0.0155	*p* = 0.0012	*p* = 0.0352	*p* = 0.0569	*p* < 0.0001	*p* = 0.0006	*p* = 0.0006
	W0 vs. W25	*p* = 0.0238	*p* = 0.0333	*p* = 0.0196	*p* = 0.0069	*p* = 0.0465	*p* = ns	*p* < 0.0001	*p* = 0.0006	*p* = 0.0023
	W0 vs. W29	*p* = 0.0728	*p* = ns	*p* = ns	*p* = 0.0098	*p* = ns	*p* = ns	*p* = 0.0009	*p* = 0.0006	*p* = 0.0008
	W1 vs. W4	*p* = 0.0124	*p* = 0.0165	*p* = 0.0382	*p* = 0.0020	*p* = 0.0563	*p* = 0.0506	*p* = 0.0175	*p* = 0.0006	*p* = 0.0080
	W1 vs. W12	*p* = 0.0008	*p* = 0.0063	*p* = 0.0326	*p* = 0.0118	*p* = 0.0084	*p* = 0.0138	*p* = 0.0027	*p* = 0.0133	*p* = 0.0071
	W1 vs. W24	*p* = 0.0563	*p* = ns	*p* = ns	*p* = 0.0552	*p* = 0.0563	*p* = ns	*p* = 0.0016	*p* = 0.0133	*p* = 0.0030
	W1 vs. W25	*p* = ns	*p* = ns	*p* = ns	*p* = ns	*p* = 0.0382	*p* = ns	*p* = 0.0046	*p* = 0.0509	*p* = 0.0080
	W1 vs. W29	*p* = ns	*p* = ns	*p* = ns	*p* = ns	*p* = 0.0542	*p* = ns	*p* = ns	*p* = 0.0353	*p* = 0.0071
	W4 vs. W12	*p* = 0.0040	*p* = 0.0109	*p* = 0.0062	*p* = 0.0558	*p* = 0.0077	*p* = 0.0382	*p* = 0.0533	*p* = ns	*p* = ns
	W4 vs. W24	*p* = ns	*p* = ns	*p* = 0.0521	*p* = ns	*p* = ns	*p* = ns	*p* = ns	*p* = ns	*p* = ns
	W4 vs. W25	*p* = ns	*p* = ns	*p* = ns	*p* = ns	*p* = ns	*p* = ns	*p* = ns	*p* = ns	*p* = ns
	W4 vs. W29	*p* = ns	*p* = ns	*p* = ns	*p* = ns	*p* = ns	*p* = 0.0158	*p* = ns	*p* = ns	*p* = ns
	W12 vs. W24	*p* = ns	*p* = ns	*p* = 0.0124	*p* = ns	*p* = ns	*p* = 0.0548	*p* = ns	*p* = ns	*p* = ns
	W12 vs.W25	*p* = ns	*p* = ns	*p* = ns	*p* = ns	*p* = ns	*p* = 0.0145	*p* = ns	*p* = ns	*p* = ns
	W12 vs. W29	*p* = ns	*p* = ns	*p* = 0.0268	*p* = ns	*p* = ns	*p* < 0.0037	*p* = ns	*p* = ns	*p* = ns
	W24 vs. W25	*p* = ns	*p* = ns	*p* = ns	*p* = ns	*p* = ns	*p* = ns	*p* = ns	*p* = ns	*p* = ns
	W24 vs.W29	*p* = ns	*p* = 0.031	*p* = ns	*p* = ns	*p* = ns	*p* = ns	*p* = ns	*p* = ns	*p* = 0.0353
	W25 vs. W29	*p* = 0.0223	*p* = ns	*p* = ns	*p* = ns	*p* = ns	*p* = ns	*p* = ns	*p* = ns	*p* = ns
**B. Secondary RP patients (SSc) treated with aminaphtone (35 patients)**		
**Timing**	**Week 0**	57.1 ± 15.7	52.2 ± 35.0	57.0 ± 22.5	42.1 ± 30.2	107.2 ± 48.1	125.6 ± 33.3	7.3 ± 1.7	2.54 ± 0.74	21.4 ± 8.5
	**Week 1**	94.8 ± 20.1	86.8 ± 41.3	80.9 ± 22.5	66.6 ± 27.1	120.2 ± 52.5	138.9 ± 40.9	5.8 ± 1.2	1.91 ± 0.92	14.9 ± 5.7
	**Week 4**	108.8 ± 25.1	92.2 ± 19.5	88.54 ± 20.7	69.8 ± 14.7	128.5 ± 48.9	145.2 ± 33.9	4.1 ± 1.4	1.29 ± 0.62	8.3 ± 4.3
	**Week 12**	102.6 ± 26.7	91.8 ± 16.8	90.1 ± 16.6	74.0 ± 13.7	138.4 ± 41.4	153.3 ± 34.3	3.2 ± 1.3	1.18 ± 0.46	5.6 ± 2.7
	**Week 24**	103.81 ± 27.6	87.9 ± 22.8	78.2 ± 20.5	71.0 ± 16.9	135.5 ± 43.2	138.3 ± 33.7	3.5 ± 0.9	1.24 ± 0.74	5.2 ± 2.5
	**Week 25**	87.5 ± 23.3	78.1 ± 25.1	70.0 ± 21.6	64.1 ± 18.1	139.1 ± 48.0	133.0 ± 29.9	3.2 ± 1.2	1.24 ± 0.74	5.8 ± 2.8
	**Week 29**	78.5 ± 18.5	78.5 ± 38.3	63.7 ± 18.8	61.8 ± 27.0	127.7 ± 39.7	130.6 ± 27.3	3.9 ± 1.2	1.32 ± 0.64	8.2 ± 4.1
**Statistical significance**	W0 vs. W1	*p* < 0.0001	*p* < 0.0001	*p* < 0.0001	*p* < 0.0001	*p* = 0.0516	*p* = 0.0176	*p* < 0.0001	*p* < 0.0001	*p* < 0.0001
	W0 vs. W4	*p* < 0.0001	*p* < 0.0001	*p* < 0.0001	*p* < 0.0001	*p* = 0.0244	*p* = 0.0005	*p* < 0.0001	*p* < 0.0001	*p* < 0.0001
	W0 vs. W12	*p* < 0.0001	*p* < 0.0001	*p* < 0.0001	*p* < 0.0001	*p* = 0.0026	*p* < 0.0001	*p* < 0.0001	*p* < 0.0001	*p* < 0.0001
	W0 vs. W24	*p* < 0.0001	*p* < 0.0001	*p* = 0.0003	*p* < 0.0001	*p* = 0.0137	*p* = 0.0205	*p* < 0.0001	*p* < 0.0001	*p* < 0.0001
	W0 vs. W25	*p* < 0.0001	*p* < 0.0001	*p* < 0.0078	*p* = 0.0001	*p* = 0.0096	*p* = 0.0544	*p* < 0.0001	*p* < 0.0001	*p* < 0.0001
	W0 vs. W29	*p* < 0.0001	*p* = 0.0002	*p* = ns	*p* = 0.0003	*p* = 0.0524	*p* = ns	*p* < 0.0001	*p* < 0.0001	*p* < 0.0001
	W1 vs. W4	*p* < 0.0001	*p* = 0.0005	*p* = 0.0007	*p* = 0.0039	*p* = 0.0037	*p* = 0.0048	*p* < 0.0001	*p* < 0.0001	*p* < 0.0001
	W1 vs. W12	*p* < 0.0001	*p* = 0.0024	*p* = 0.0016	*p* = 0.0003	*p* = 0.0005	*p* < 0.0001	*p* < 0.0001	*p* < 0.0001	*p* < 0.0001
	W1 vs. W24	*p* = 0.0548	*p* = ns	*p* = ns	*p* = ns	*p* = ns	*p* = ns	*p* < 0.0001	*p* < 0.0001	*p* < 0.0001
	W1 vs. W25	*p* = ns	*p* = ns	*p* = ns	*p* = ns	*p* = 0.0526	*p* = ns	*p* < 0.0001	*p* < 0.0001	*p* < 0.0001
	W1 vs. W29	*p* = 0.0010	*p* = ns	*p* = 0.0014	*p* = ns	*p* = ns	*p* = ns	*p* < 0.0001	*p* < 0.0001	*p* < 0.0001
	W4 vs. W12	*p* = ns	*p* = ns	*p* = ns	*p* = 0.0015	*p* = 0.0154	*p* = 0.0011	*p* < 0.0001	*p* = 0.0437	*p* = 0.0002
	W4 vs. W24	*p* = ns	*p* = ns	*p* = ns	*p* = ns	*p* = ns	*p* = ns	*p* = 0.0157	*p* = ns	*p* = 0.0001
	W4 vs. W25	*p* < 0.0001	*p* = 0.0065	*p* = 0.0003	*p* = ns	*p* = ns	*p* = 0.0381	*p* < 0.0001	*p* = ns	*p* = 0.0007
	W4 vs. W29	*p* < 0.0001	*p* = ns	*p* < 0.0001	*p* = ns	*p* = ns	*p* = 0.0253	*p* = ns	*p* = ns	*p* = ns
	W12 vs. W24	*p* = 0.0196	*p* = ns	*p* = 0.0010	*p* = ns	*p* = ns	*p* = 0.0338	*p* = ns	*p* = ns	*p* = ns
	W12 vs.W25	*p* < 0.0001	*p* = 0.0014	*p* < 0.0001	*p* = 0.0079	*p* = ns	*p* = 0.0018	*p* = ns	*p* = ns	*p* = ns
	W12 vs. W29	*p* < 0.0001	*p* = 0.0434	*p* < 0.0001	*p* = 0.0224	*p* = ns	*p* = 0.0014	*p* = 0.0003	*p* = 0.0230	*p* = 0.0003
	W24 vs. W25	*p* < 0.0001	*p* = 0.0002	*p* = 0.0066	*p* = 0.0069	*p* = ns	*p* = ns	*p* = ns	*p* = ns	*p* = ns
	W24 vs.W29	*p* < 0.0001	*p* = ns	*p* < 0.0001	*p* = 0.0395	*p* = ns	*p* = ns	*p* = 0.0472	*p* = ns	*p* = 0.0005
	W25 vs. W29	*p* < 0.0008	*p* = ns	*p* = 0.0140	*p* = ns	*p* = 0.0336	*p* = ns	*p* < 0.0001	*p* = ns	*p* = 0.0006


*Variation of blood perfusion (BP), Raynaud Condition Score (RCS), Raynaud’s frequency (number of daily attacks) and duration (minutes) from baseline (W0) to week twenty-four (W24) during aminaphtone treatment, and after one (W25) and five (W29) weeks since treatment discontinuation, in patients with primary **(A)** or secondary **(B)** Raynaud’s phenomenon (RP). PU = perfusion units. SSc = systemic sclerosis. Statistical significance between single times: Wilcoxson test*.

The results concerning clinical efficacy were similar in both primary and secondary RP patients (see Tables 3A,B for further details), as well as in patients with limited or diffuse skin disease, and in patients with “Early,” “Active,” or “Late” NVC pattern of microangiopathy.

Any statistically significant change as in blood perfusion, as in clinical symptoms (RCS, frequency and duration of Raynaud’s attacks), was not observed in the control group of RP patients (either primary or secondary RP) between W0 and W24 (see Table 2B for perfusion and clinical values).

One and five weeks after treatment discontinuation, a progressive reduction of skin blood perfusion was recorded (see Table 2A for statistical details). However, five weeks after aminaphtone discontinuation blood perfusion values were yet significantly higher than those at baseline in the majority of skin areas (Table 2A). Also clinical efficacy was still sustained 5 weeks after treatment discontinuation (Table 2A).

Serious adverse events were not observed during the study. Aminaphtone was stopped in two patients due to headache, which recovered one days after treatment discontinuation. One patient was lost during follow-up. Blood cell count, liver aminotransferase, and creatinine values were also routinary assessed every 3 months and no abnormal variation of these parameters was observed.

## Discussion

This is the first feasibility study that has evaluated the effects of aminaphtone treatment on both skin blood perfusion and clinical symptoms in patients affected by either primary or secondary RP.

The study demonstrates that aminaphtone treatment increases in short-time skin blood perfusion at the level of hands and face, as well as ameliorates RP clinical symptoms, with a sustained efficacy until 6 months. The results were similar for both primary and secondary RP patients. Of interest, any statistically significant difference was not observed concerning skin blood perfusion and RP clinical improvement between patients with different pattern of nailfold microangiopathy (“early,” “active,” or “late”), as well as between lcSSc and dcSSc patients, supporting the clinical efficacy of aminaphtone in different subgroups of RP patients (Sulli et al., 2017). Furthermore, skin blood perfusion increased after aminaphtone treatment also at the level of face, which usually shows similar blood perfusion values as in SSc patients as in healthy subjects (Sulli et al., 2014; Ruaro et al., 2016).

This study demonstrates also a progressive statistically significant improvement of the RCS, frequency and duration of RP attacks from baseline to 12 weeks of treatment. Similar results have been highlighted even by other studies where endothelin receptor antagonists or phosphodiesterase 5 inhibitors were administered to patients with secondary RP (Selenko-Gebauer et al., 2006; García de la Peña-Lefebvre et al., 2008; Roustit et al., 2013; Kamata and Minota, 2014; Lee et al., 2014).

Raynaud’s phenomenon significantly impacts on quality of life in all subjects. It provokes the deterioration of patient quality of life, not only in terms of pain, but also due to the extreme difficulty in performing normal daily activities. An international survey involving 443 people with self-reported RP showed that 64% had poor ability to control their attacks and only 16% believed that one current medication was effective (Hughes et al., 2015). Treatments were generally considered tolerable but seldom fully effective, and the results confirmed an unmet need for new treatments, as the approach to the management of the disorder was based on published information, expert opinion, and current practices (Hughes et al., 2015).

Current treatments for RP include calcium channel blockers, i.v. prostanoids, and topical glyceryl trinitrate (applied locally to the digits), while key strategic treatment are the increased use of phosphodiesterase type V inhibitors in severe RP (Kowal-Bielecka et al., 2017). Other treatments being researched include botulinum toxin (for severe digital ischemia/ulceration), and several other drugs including oral prostanoids (Shah et al., 2013; Fardoun et al., 2016; Motegi et al., 2016; Żebryk and Puszczewicz, 2016). In view of costs and feasibility, the experts in EULAR propositions recommended that calcium antagonists were first-line therapy in the treatment of secondary RP in SSc, and intravenous prostanoids were recommended when calcium antagonists had failed. As both types of drugs may induce side effects of vascular origin, the experts recommend particular attention if prostanoids are combined with calcium antagonists (Kowal-Bielecka et al., 2009).

Recently, increased availability and interest in nailfold capillaroscopy and laser technologies, by assessing morphological and functional capillary/microcirculatory variations, paves the way for studies on early intervention and vascular protection in RP/SSc patients (Cutolo et al., 2010a,b, 2013, 2014; Rosato et al., 2010; Ruaro et al., 2014).

Our results highlight the effectiveness of aminaphtone in the treatment of RP. An interesting observation, not reported among the results of the study, was that some patients with oedematous/puffy fingers (8 patients complaining of primary RP, and 6 patients affected by secondary RP to SSc; among these 5 with the Early and 1 with the Active pattern of microangiopathy) reported an improvement, until complete resolution of symptoms, during treatment with aminaphtone. This was possibly related to the reduction of the oedematous phase that this molecule has been shown to induce in several studies (De Anna et al., 1989; Martinez-Zapata et al., 2016).

The mechanism of actions of aminaphtone is unclear. Recent studies have reported that aminaphtone reduces vessel permeability and tissue oedema (De Anna et al., 1989; Scorza et al., 2008a,b). Furthermore, results from *in vitro* studies suggest that among different mechanisms of action, aminaphtone may down-regulate the E-selectin (ELAM-1), Vascular Cell Adhesion Molecule-1 (VCAM-1) and Intra Cellular Adhesion Molecule-1 (ICAM-1) expression, as well as cytokine/chemokine and endothelin-1 production on cultured human endothelial cells (Scorza et al., 2008a,b; Salazar et al., 2016). Of note, endothelin-1 generates vasoconstriction on microvessels and its serum level is increased in both patients with primary and secondary RP (Sulli et al., 2009).

Aminaphtone down-regulates both gene transcription and protein production of almost all the most important molecules responsible for the inflammatory state, including IL-6 (Salazar et al., 2016). It also decreases the levels of TGF-β which can lead to pulmonary fibrosis by activating the fibroblasts to produce an excessive deposition of collagen (Salazar et al., 2016).

Probably all these actions of aminaphtone support the first results of Parisi et al. (2015) who reported the possible effectiveness of aminaphtone in association with standard therapy in RP patients, resulting in a synergic effect on vasospastic phenomena.

**FIGURE 3:**
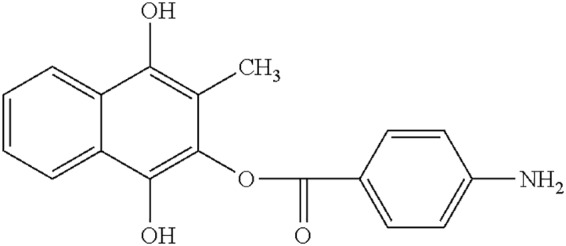
Structure formula of aminaphtone (C18-H15-N-O4).

As reported inside technical sheet, concerning pharmacokinetic properties, administered to humans aminaphtone is partially metabolized to phthiocol and eliminated through the urine within the 72nd hour. The maximum excretion level was observed to be 6 h after administration. Concerning preclinical safety data, the tests of acute toxicity (4 animal species for doses up to 3 g/kg), subacute toxicity (2 animal species up to 100 mg/kg, for 90 days) and chronic toxicity (50 mg/kg in the dog for 280 days) showed no symptoms of tissue lesions or changes in organ functions. Aminaphtone also had no teratogenic or mutagenic effects. The structure formula of aminaphtone is reported in Figure 3.

This study has some limitations. It’s not randomized, not blind, and underpowered to detect small treatment effects. However, the high statistical significance of the results despite the small cohort of enrolled patients suggests the possibility to confirm these results by performing larger randomized clinical studies. The two patient groups cannot be completely compared: blood perfusion was assessed only at basal time and after 6 months in the control group, as the decision of including a control group into this pilot study came later, after enrolment completion. In order to assess the trend of blood perfusion during the same months of the year in both patient groups, a control group of patients who performed the first LASCA evaluation in the same month of the aminaphtone-treated group was enrolled. Also the possible effect of aminaphtone on digital ulcer healing/prevention was not assessed in this study, as it was not addressed to this endpoint. In both groups, aminaphtone and control group, only one patient developed a new digital ulcer; however, patients in the control group were showing a less aggressive disease at baseline. Despite this, aminaphtone treatment was found effective in increasing skin BP and ameliorating RP clinical symptoms, while any modification of skin BP was not detected in the untreated group of patients. Another points is the nature of this uncontrolled clinical study that does not provide the sureness that all patients have taken two tablets of aminaphtone per day for six months (the treatment cost is covered by patient in our country): at best of our knowledge, the patients declared adherence to the treatment, but this may not be proven and the eventuality might be the cause of a slight reduction of blood perfusion after week 12 of treatment. Finally, the seasonal variation of temperature should not have influenced the results of the study, as all patients were enrolled in November, and the study carried out during the six colder months of the year.

By considering recent data showing that 5–15% of patients diagnosed as affected by primary RP may shift to secondary RP during follow-up, the possible role of aminaphtone in the prevention of this transition should be longitudinally investigated (Cutolo et al., 2007a; Ingegnoli et al., 2010; Bernero et al., 2013; Trombetta et al., 2016; Gualtierotti et al., 2017).

## Conclusion

Aminaphtone treatment was well tolerated and improved in short time skin blood perfusion and RP clinical symptoms, with a sustained efficacy until six months. A randomized, blind, controlled, clinical trial including a larger number of subjects is advisable to confirm these early results and to assess the possible role of aminaphtone also in the treatment/prevention of other SSc-related clinical manifestations.

## Data Availability

The datasets generated and/or analyzed during the current study are not publicly available for ethical and privacy reasons, but are available from the corresponding author on reasonable request.

## Author Contributions

BR and AS were involved in the conception and design of the study, acquisition of data, basic analysis and interpretation of data, drafting of the manuscript, and revising it critically for important intellectual content. CP, SP, and EA were involved in the acquisition of data, basic analysis and interpretation of data, drafting of the manuscript, and revising it critically for important intellectual content. AS performed the statistical analysis. All authors read and approved the final manuscript.

## Conflict of Interest Statement

AS declares an unconditioned research grant from Laboratori Baldacci. The remaining authors declare that the research was conducted in the absence of any commercial or financial relationships that could be construed as a potential conflict of interest.
